# Resection of urachal anomalies in dogs with recurrent lower urinary tract disease

**DOI:** 10.1111/vsu.13311

**Published:** 2019-08-14

**Authors:** Judith Visser, Anne Kummeling, Marjon A. van Nugteren, Guy C. M. Grinwis, Bouvien A. W. Brocks

**Affiliations:** ^1^ Department of Companion Animal Medicine Utrecht University Utrecht The Netherlands; ^2^ Department of Pathobiology Utrecht University Utrecht The Netherlands

## Abstract

**Objective:**

To determine whether surgical removal of urachal anomalies improves the outcomes of dogs with recurrent lower urinary tract disease (LUTD) and bacterial urinary tract infection (BUTI).

**Study design:**

Retrospective study.

**Animals:**

Thirty‐three dogs with urachal anomalies and recurrent LUTD or BUTI.

**Methods:**

Medical records of dogs with LUTD or BUTI and a diagnosis of urachal anomaly treated by partial cystectomy were reviewed. A minimum follow‐up of 9 months was required for inclusion.

**Results:**

Median age at onset of clinical signs was 12 months (range, 1 month to 10 years). Urachal anomalies were detected with histopathology in 20 of 28 (71%) dogs. At a median follow‐up of 22 months (range, 9‐114), 21 of 28 (64%) dogs were free of signs of LUTD. Nine (27%) dogs exhibited reduced signs of LUTD; in three (9%) dogs, no clinical improvement was observed. Among the 25 dogs with confirmed preoperative BUTI, 22 clinically improved with surgery.

**Conclusion:**

Partial cystectomy reduced the long‐term severity of clinical signs and risk of recurrence of LUTD or BUTI in dogs with confirmed or suspected urachal anomalies.

**Clinical significance:**

Partial cystectomy should be considered as an adjunct to the treatment of LUTD and BUTI in dogs.

## 1 INTRODUCTION

Lower urinary tract disease (LUTD) in dogs is predominantly caused by bacterial urinary tract infection (BUTI), and the most common pathogen isolated in BUTI is *Escherichia coli*.^1^ It can recur, despite appropriate antimicrobial treatment, due to predisposing factors such as endocrine disease, immunosuppression, urinary stasis due to abnormal micturition, anatomic anomalies, pyelonephritis, prostatitis, uroliths, or neoplasia.^2^ Although congenital anatomic urachal anomalies have been identified in dogs,^3^ their association with recurrent BUTI is currently unknown.

**Figure 1:**
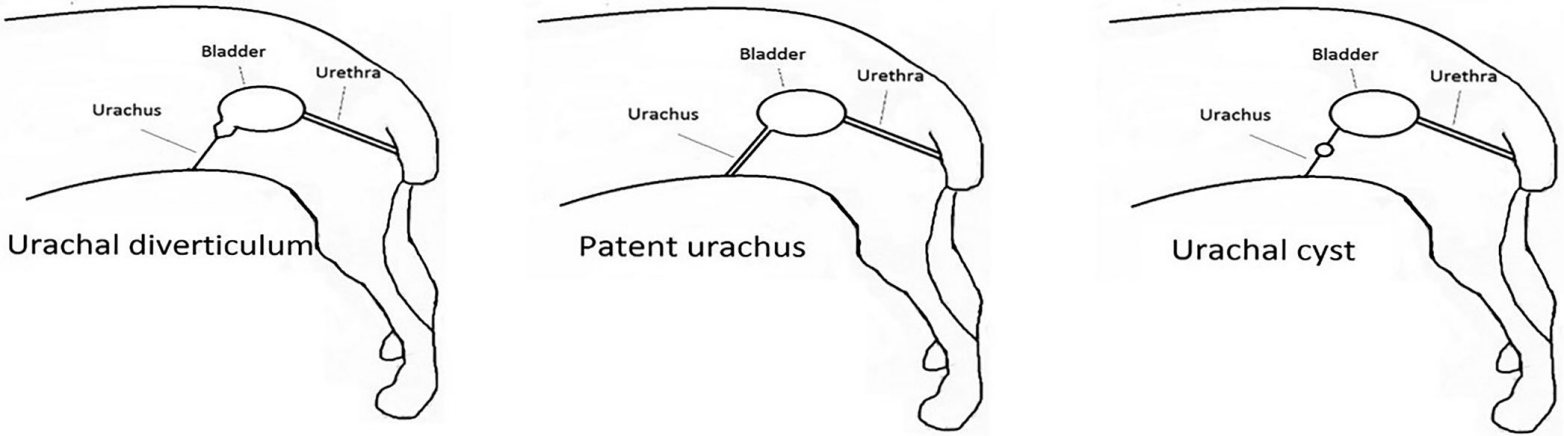
Three urachal anomaly types described anatomically in dogs^7^

Urachal anomalies may predispose animals to recurrent urinary tract infections due to urinary stasis and reduced clearance during normal micturition,^4^ possibly because of the more ventral and cranial position of the bladder due to the attachment of the urachal anomaly to the cranial apex of the bladder.^5^ Urachal anomalies are described as macroscopic diverticula in the urothelial lining that protrude the mucosa and submucosa through the muscular layering of the bladder wall, creating small lesions that may be detectable only by histology.^6^ Four types of congenital urachal anomalies that can persist after birth have been identified, and three of these have been reported in dogs (Figure 1).^7^ The fourth type, urachal sinus, has been identified in man only.^8^ Patent urachus has been reported in dogs^9^ as well as in cats,^10^ horses,^11^ cows,^12^ and more exotic species such as walruses^13^ and rhinoceros.^14^ Other reported types are vesicourachal diverticula in dogs,^3^ cats,^15^ and guinea pigs^16^ and urachal cysts in dogs^3^ and cows.^17^ Vesicourachal diverticula are most frequently seen in dogs^3^, ^18^ and can differ in size. Some vesicourachal diverticula are too small to be identified with advanced diagnostic imaging. They consist of small areas of urothelium (transitional cell epithelium) with a microscopic lumen, which can be diagnosed with histopathological examination if assessed at the exact location. The clinical significance of microscopically small vesicourachal diverticula is unknown.^3^ Macroscopically visible vesicourachal diverticula can usually be identified by advanced diagnostic imaging or cystoscopy (Figure 1).^19^

The prevalence of urachal anomalies was found to be 34% (17 diverticula and one cyst) in a 2005 study of 50 dogs without clinical evidence of urinary tract infection.^3^ The prevalence of urachal anomalies in feline cadavers with an unknown history was 24% (179/735) in another study.^5^ The second part of that study consisted of cats with feline urinary syndrome, and removal of urachal anomalies reportedly resulted in a lower recurrence rate (8/49 cats that underwent surgical resection of the lesion vs 27/48 cats without surgery).^5^ A single case report in 1981 described a cat with persistent urinary tract infection that was treated surgically by resection of a urachal anomaly. Surgery resulted in the resolution of the infection, but the duration of follow‐up was not reported.^15^

Surgical excision was previously described as a successful treatment modality for a patent urachus in dogs,^9^ cats,^5^, ^19^ and cows.^20^ However, clinical signs and diagnosis of a patent urachus differ from the other types of urachal anomalies. Surgical removal of an acquired diverticulum of the bladder in two dogs with recurrent BUTI has been described^21^, ^22^; however, these diverticula were not located at the apex and were not associated with a urachal remnant. Partial cystectomy resulted in the resolution of clinical signs in these cases. Nine dogs with signs of LUTD had vesicourachal diverticula that were surgically resected and showed clinical signs of hematuria and/or pollakiuria for 2 to 48 months prior to surgery in a 1979 study conducted by Wilson et al.^23^ Although only two dogs had a follow‐up longer than 2 months in that study, one had clinical recurrence 10 months after surgery, and the other had a positive culture result 18 months after surgery.

It is unclear whether removal of urachal anomalies is required or beneficial in dogs with LUTD and/or BUTI to prevent recurrence. The objective of this retrospective study was to determine whether partial cystectomy to resect urachal anomalies improves the long‐term outcome of recurrent LUTD or BUTI in dogs. We hypothesized that surgical resection reduces clinical signs and prevents recurrence of LUTD in dogs with urachal anomalies.

## 2 MATERIALS AND METHODS

### 2.1 Patient selection

The medical records of client‐owned dogs that had been referred to the veterinary teaching hospital of the Utrecht University between October 1, 2007 and December 1, 2016 were reviewed. In total, 146 dogs with a history of clinical signs consistent with LUTD were identified. Among these, only those in which a urachal anomaly was diagnosed and that underwent a partial cystectomy were included in the study.

### 2.2 Diagnostic procedures

Before surgery, a standardized urological diagnostic examination was recommended, including physical examination, abdominal ultrasonography, ultrasound‐guided cystocentesis, urinalysis, and urine culture. Urachal anomaly was diagnosed by ultrasonography, positive‐contrast retrograde cystography, cystoscopy, or evaluation of the cranial apex of the bladder during surgery. Cystoscopy was performed with a rigid, 30°‐view scope with a 1.9‐mm, 2.7‐mm, or 4.0‐mm diameter, depending on size of the dog (Karl Storz, Netherlands), via the urethra in female dogs and via minimal parapreputial celiotomy and cystotomy in male dogs. When a urachal anomaly was suspected but could not be visualized with these techniques, an explorative cystotomy was discussed with the owner as a next diagnostic step.

### 2.3 Surgical technique

Partial cystectomy was recommended to the owners of all dogs with a urachal anomaly. Dogs were anesthetized with an individualized anesthetic protocol by a board‐certified veterinary anesthesiologist. Surgery was performed either immediately after cystoscopy or at a later time, depending on owner and surgeon preference.

Surgery was performed by a board‐certified surgeon or a surgical resident under direct supervision of a board‐certified surgeon. The caudal abdomen was approached via standard caudal midline celiotomy. The bladder was localized, and the cranial apex was identified. Three partial thickness stay sutures were placed. One stay suture was placed in the apex of the bladder where the presumed urachal remnant was localized, and the remaining two stay sutures were placed on both lateral sides of the bladder for stabilization. Cranial traction was applied to the first stay suture to exteriorize the bladder. When the location of the urachal anomaly could be visualized from the serosal or mucosal side of the bladder wall, it was macroscopically resected with a margin of approximately 1 cm around the anomaly. When the anomaly could not be visualized, the apex of the bladder was removed at the most cranial tip of the bladder at the location of the presumed anomaly.

The bladder defect was closed in a double layer and one continuous full‐thickness appositional layer followed by one continuous inverting seromuscular layer with monofilament absorbable suture, and the abdomen was closed routinely. Postoperatively, the dogs were treated with a nonsteroidal anti‐inflammatory drug for 7 days (carprofen, 2 mg/kg orally every 12 hours) and tramadol for 3 days (2‐5 mg/kg orally every 8 hours). The excised tissue was fixed in a 4% neutral‐buffered formaldehyde solution and submitted for histopathological examination. The fixed tissues were trimmed and routinely embedded in paraffin, and 4‐μm tissue sections were stained with hematoxylin and eosin for histopathological evaluation by a board‐certified veterinary pathologist.

### 2.4 Follow‐up

Standard recommended follow‐up consisted of clinical examination as well as recheck abdominal ultrasonography and urine culture at 3 to 4 weeks after surgery. A standardized questionnaire (Appendix S1) was completed by the owners via telephone or email contact at a minimum of 9 months after surgery. The owners were asked whether the clinical signs of LUTD in their dogs had improved or resolved after surgery (scale of 0‐5, with 5 as most severe) and how satisfied they were with the long‐term outcome of surgery (scale 0‐5, with 5 as very satisfied). The owners were also asked whether they had visited a veterinarian because of presumed recurrence of LUTD in the dog, whether a culture had been repeated, and whether their dog had been treated with antibiotics or other medication since the last visit.

## 3 RESULTS

In total, 33 dogs met the inclusion criteria, 17 males (seven intact and 10 neutered) and 16 females (eight intact and eight spayed). Breeds included English cocker spaniel (n = 3), Bernese mountain dog (n = 3), Staffordshire bullterrier (n = 2), Cavalier King Charles spaniel (n = 2), boxer (n = 2), labradoodle (n = 2), and one each of Newfoundland dog, Great Dane, Chihuahua, chow chow, English springer spaniel, German shepherd, Rottweiler, Dutch sheepdog, Labrador retriever, French bulldog, petit basset griffon, Irish Terrier, shepherd cross, Airedale terrier, cane corso, Swiss mountain dog, Rhodesian ridgeback, Weimaraner, and bearded collie. Median age at onset of clinical signs of LUTD was 12 months (range, 1 month to 10 years). The most common clinical signs observed were pollakiuria (19/33 [58%]), stranguria (19/33 [58%]), hematuria (15/33 [45%]), and active incontinence (10/33 [30%]). Most dogs presented with more than one clinical sign. The median duration of clinical signs before surgery was performed was 8 months (range, 1 month to 8 years). The owners (n = 16) rated the severity of the clinical signs on a scale of 0 to 5 (0, no clinical signs to 5, most severe); five (49%) dogs were rated as 5; 12 (36%) dogs were rated as 4, and five (15%) dogs were rated as 3.

Among the 33 dogs that were included, 28 had one or more urine cultures performed before surgery. Among these, 25 (89%) had positive culture results, and three (11%) had negative culture results but showed signs of inflammation or infection according to urinalysis. One of the three dogs with a negative culture result was receiving antibiotic therapy when the urine was collected. *Escherichia coli* was the most commonly isolated bacteria and was found in 16 (64%) dogs. *Proteus mirabilis* was isolated in seven (28%) dogs. Among the five remaining dogs without a preoperative urine culture, two were determined to have LUTD by the referring veterinarian on the basis of clinical signs only, and the remaining three diagnoses of LUTD were based on clinical signs and urinalysis.

Thirty‐two dogs were treated with antibiotic regimens prior to surgery as follows (Appendix S2): one antibiotic regimen (n = 3), two regimens (n = 9), three regimens (n = 8), and more than three treatment regimens (n = 12). The most commonly prescribed antibiotic was amoxicillin‐clavulanic acid; other antibiotics included sulfamethoxazole/trimethoprim, enrofloxacin, cephalexin, doxycycline, and metronidazole. Treatment duration varied from 5 days to 6 weeks. One dog did not receive antibiotic therapy prior to surgery. This dog had shown pollakiuria, stranguria, and hematuria for 2 months and had two urine cultures performed preoperatively. Urinalysis results revealed increased leucocytes and erythrocytes according to cytology. Positive‐contrast cystography was performed, and a urachal diverticulum was diagnosed and removed. Follow‐up for this dog was 30 months, during which no recurrence of clinical signs was observed.

**Figure 2:**
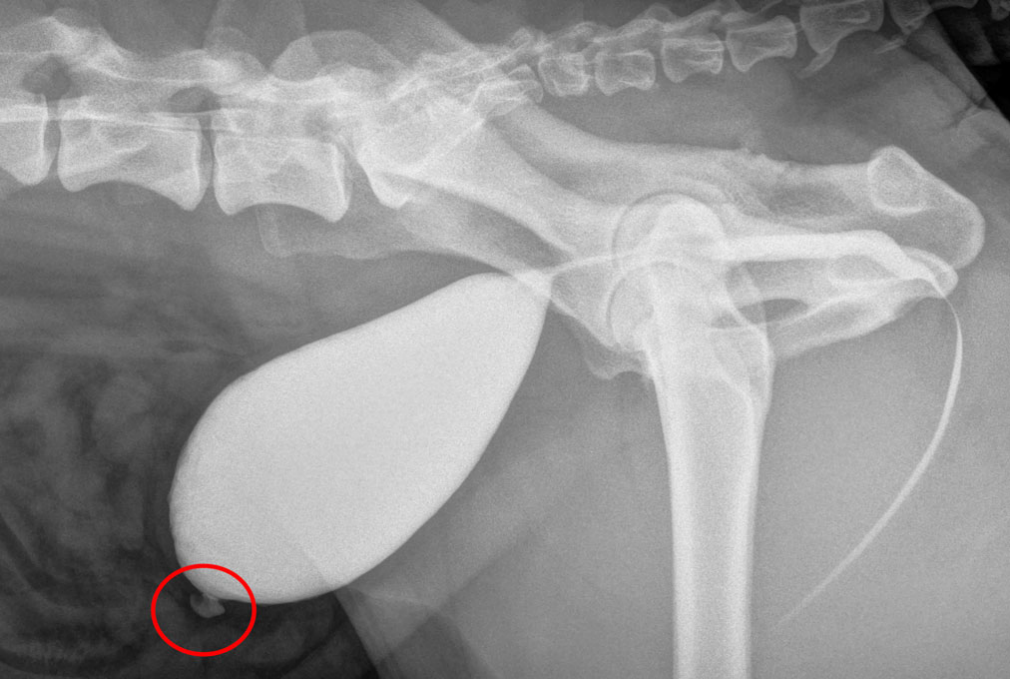
Appearance of a diverticulum on positive‐contrast cystography. The red circle marks the location of the urachal anomaly

A vesicourachal diverticulum was diagnosed in 32 dogs, and a urachal cyst was found in one dog. All dogs had ultrasonographic examination of the abdomen performed, from which the urachal anomaly was diagnosed in six dogs. Among these, one dog had a urachal cyst. Positive‐contrast cystography was performed in 11 dogs, and the anomaly was diagnosed in six dogs (Figure 2). The urachal anomaly was found during cystoscopy (Table 1) in 16 of 18 dogs.

**Table 1:** Four techniques used for identification of urachal anomalies

Technique	Procedures performed, n	Positive cases, n (%)
Ultrasound	33	6 (18)
Positive‐contrast cystography	11	6 (55)
Cystoscopy	18	16 (89)
Pathology	28	20 (71)

*Note*: Three diagnostic techniques were used preoperatively. The fourth technique was postoperative histopathological examination of excised tissue.

The overall distribution was evenly distributed across sexes (17 males and 16 females), but cystoscopy was much more often performed in female dogs (n = 15) than in male dogs (n = 3); we diagnosed the anomaly in 14 of the 15 females and two of the three males. Only one female dog had no cystoscopy performed preoperatively; the urachal anomaly was diagnosed by positive‐contrast cystography.

**Figure 3:**
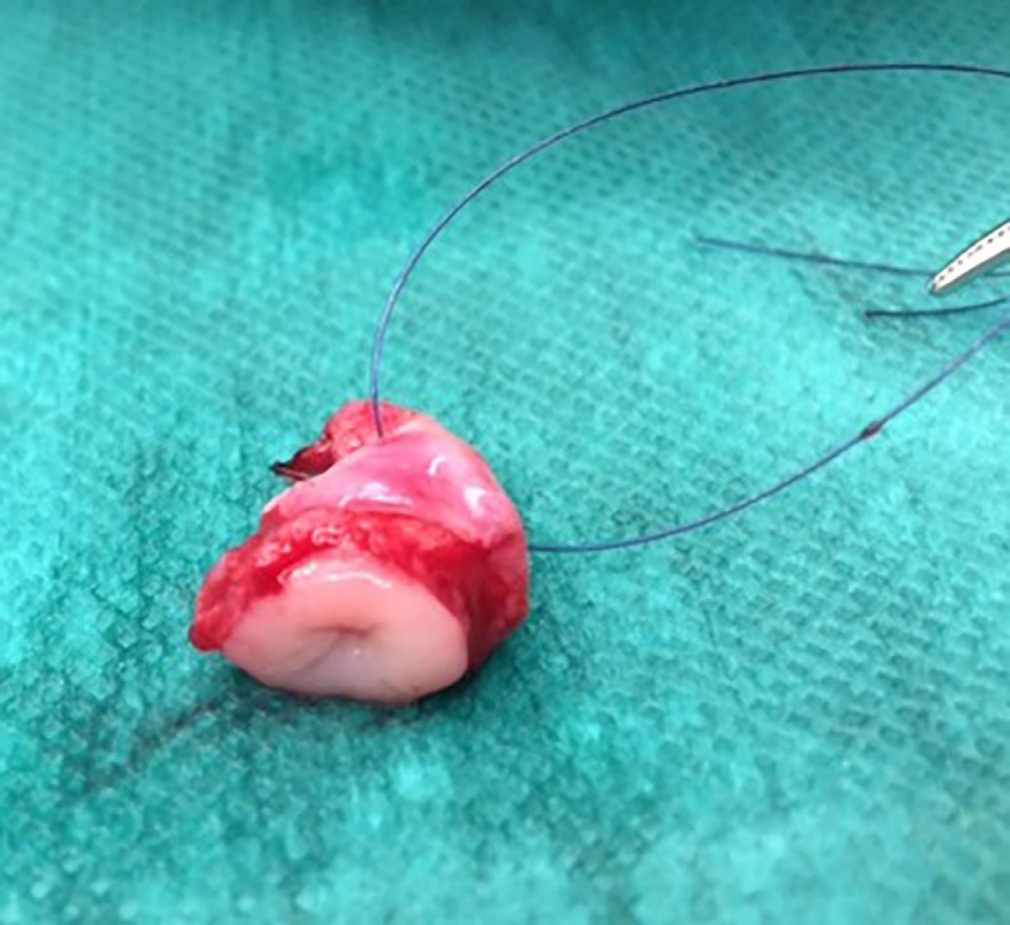
Appearance of a resected urachal diverticulum

Both positive‐contrast cystography and cystoscopy were performed in four dogs. For all of these dogs, positive‐contrast cystography failed to reveal the urachal remnant; however, the lesion was diagnosed with cystoscopy for three of these dogs. The anomaly in the fourth dog was diagnosed by surgical exploration of the bladder. In seven dogs (six males and one female), no preoperative evidence of a urachal anomaly was found. In all these dogs, ultrasonography (n = 7), positive‐contrast cystography (n = 2), or cystoscopy (n = 2) were negative (Appendix S3). A history of LUTD for 3 to 7 years was found in four of these dogs; LUTD was treated with antibiotics but recurred despite appropriate treatment. The remaining three dogs had a history of recurrent LUTD for 3, 5, or 6 months, and all had received more than three antibiotic regimens during these short timeframes. Based on the clinical history and after excluding other causes, the decision was made to surgically evaluate the cranial apex of the bladder. The urachal anomaly was visualized and surgically resected in all seven dogs (Figure 3), and its presence was confirmed by histopathological examination.

**Figure 4:**
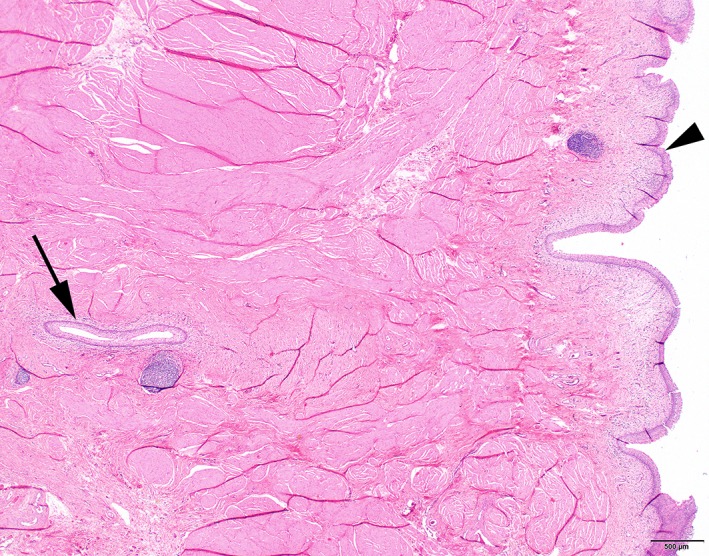
Histology of the urinary bladder of a dog at the luminal side covered with urothelium (transitional cell epithelium; arrowhead). Deep within the muscularis, a section through a tubular structure lined with urothelium is visible (arrow). Hematoxylin and eosin stain, image ×2

**Figure 5:**
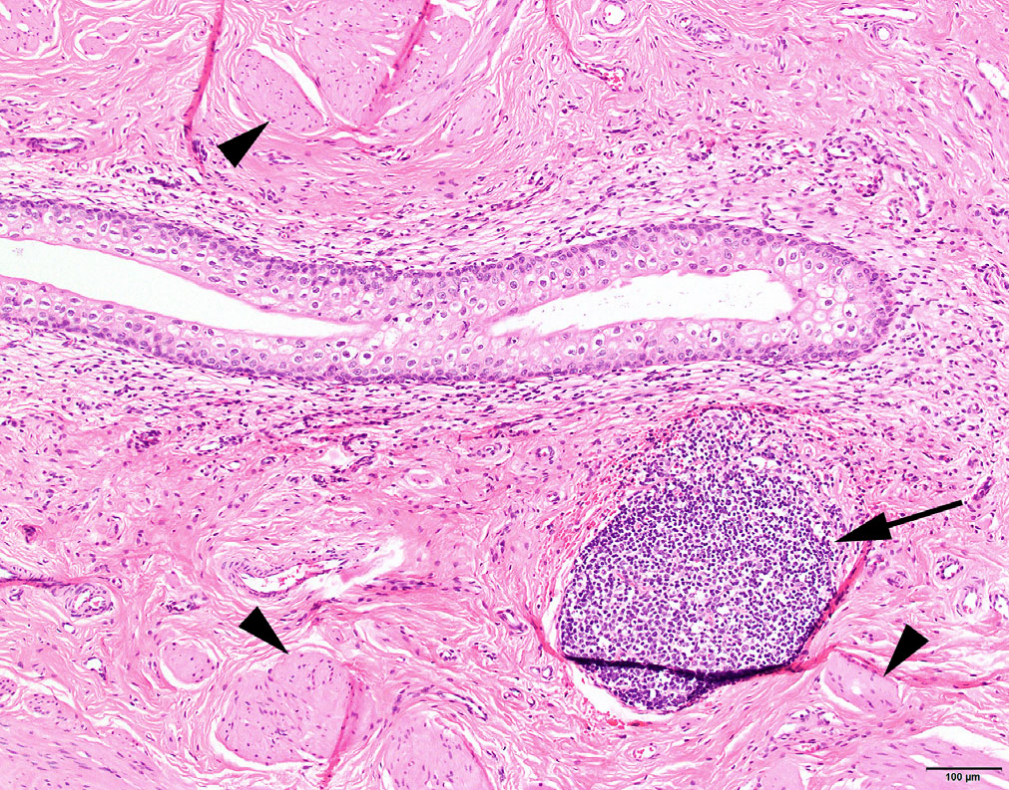
Histology of a vesicourachal diverticulum in the wall of the urinary bladder of a dog. The tubular vesicourachal diverticulum is clearly visible, surrounded by bundles of smooth muscle cells of the muscularis (arrowheads). In the subepithelial stroma, a cluster of lymphocytes is visible (arrow). Hematoxylin and eosin stain, image ×10

Histopathology of excised tissue was performed in 28 of 33 dogs (Figures 4 and 5 *)*; diagnoses were confirmed in 71% of dogs by this method—vesicourachal diverticula in 19 (68%) dogs and a urachal cyst one (3%) dog. Histopathology of vesicourachal diverticula was characterized by the presence of a tubular structure lined with urothelium, often accompanied by an inflammatory infiltrate in the lamina propria and submucosa consisting of lymphocytes, plasma cells, and macrophages.

Postoperative antibiotics were prescribed for 31 dogs for a median of 12 days (range, 6 days to 4 weeks). The most common antibiotic prescribed was amoxicillin‐clavulanic acid (27/31 dogs); the remaining four dogs were treated with enrofloxacin (three dogs, 7 days to 4 weeks) or sulfamethoxazole/trimethoprim (one dog, 4 weeks), based on culture results.

Short‐term follow‐up revealed minor complications only, as reported by the owners. Mild hematuria (n = 2), pollakiuria (n = 5), and incontinence (n = 3) were most frequently reported during the first 3 to 4 weeks after surgery. Seven dogs were prescribed oxybutynin symptomatically, which resolved clinical signs of dysuria. Twenty‐two dogs were seen for follow‐up 3 to 4 weeks after surgery; one dog was seen for follow‐up 2 months after surgery. Short‐term follow‐up was obtained by telephone only 1 to 2 weeks after surgery for seven dogs. No short‐term follow‐up was available for the remaining three dogs, but long‐term follow‐up was obtained 1 or 2 years after surgery.

No dogs were lost to long‐term follow‐up (median, 22 months; range, 9‐114). Four dogs died or were euthanized during the follow‐up period, three for reasons unrelated to urinary tract disease or surgical complications (recurrent sarcoma 12 months after surgery, refractory epilepsy 16 months after surgery, and a suspected intracranial mass 31 months after surgery). The fourth dog was euthanized 10 months after surgery for severe dysuria and tenesmus; urine culture result at 1 month after surgery was negative. Clinical signs had not improved after surgery, but no additional diagnostics were performed; therefore, no definitive diagnosis was made for this dog.

The owners of 21 dogs (nine female, 12 male) reported no recurrence of clinical signs of LUTD during the long‐term follow‐up. Among these clinically recovered dogs, 10 had negative urine culture results at 1 month after surgery to confirm the absence of BUTI. One dog had a positive urine culture result at 2 months after surgery without any clinical signs. In the remaining 11 dogs without clinical recurrence of LUTD, no follow‐up urine culture was performed, so the absence of BUTI could not be confirmed.

Nine dogs (six female, three male) had less severe or less frequent clinical signs of LUTD after surgery. Among these dogs, three had no urine culture performed postoperatively. The remaining five dogs each had one episode of BUTI confirmed by positive urine culture results (performed at 1 month, 2 months, 6 months [2 dogs], or 10 months after surgery). In three of these dogs, clinical signs resolved, and bacterial clearance was confirmed by a negative culture result after appropriate antibiotic treatment. However, in one of these dogs, clinical signs still recurred after antibiotic treatment was discontinued, despite the absence of bacteria.

For the remaining three dogs (two males and one female), no improvement was reported in long‐term severity of clinical signs, but, in two dogs, no additional urine cultures were performed. In the third dog, the result of a urine culture performed at 1 month after surgery was negative. As described earlier, this dog was euthanized at 10 months after surgery.

Overall, 91% (30/33) of the dogs in this study clinically improved after surgery. From the subgroup of 25 dogs with LUTD that had a confirmed preoperative BUTI, 22 (88%) owners reported clinical improvement after surgery. On a scale of 0 to 5 (5 = most severe), 21 (64%) owners rated severity of clinical signs of their dog after urachal resection as 0 or nonexistent, one (3%) owner rated clinical signs as 1, two (6%) owners rated clinical signs as 2, four (12%) owners rated clinical signs as 3, three (9%) owners rated clinical signs as 4, and two (6%) owners rated clinical signs as 5. Both dogs rated as 5 after surgery had been rated as 5 prior to surgery as well. Long‐term owner satisfaction (scale 0‐5, with 5 as very satisfied) with the clinical outcome of surgery was excellent in 70% (23/33) of dogs. Satisfaction was rated as 4 by four (4/33 [12%]) owners and as three (2/33 [6%]) by two owners. Three owners (3/33, 9%) were very unsatisfied with the procedure, rating it as 0. In one dog, owner satisfaction was unknown.

For eight dogs, results of histopathology failed to confirm the diagnosis of a urachal anomaly. For seven of these, intraoperative visualization of an abnormality in the apex of the bladder was described. For the eighth dog, intraoperative visualization of the lesion was not clearly described in the surgery report, but the diagnosis was made by cystoscopy. Among these eight dogs, four had a recurrence of clinical signs. Five dogs were determined to have a urachal anomaly by cystoscopy. In the remaining three dogs, the diagnosis was confirmed by positive‐contrast cystography (n = 2) or ultrasound (n = 1).

No histopathological examination of excised tissue was performed for five dogs. One of these showed an improvement in severity of clinical signs but had an episode of BUTI 2 months after surgery. The diagnosis in this dog was made by positive‐contrast cystography. In the five dogs without a histopathological confirmation of the urachal anomaly and with recurrence of clinical signs, an ultrasound performed at 1 month (three dogs) or 2 months (two dogs) after surgery did not reveal a urachal anomaly. No additional diagnostics were performed in these cases.

Cultures were performed postoperatively in 16 of 33 dogs, and results were negative in 10 (63%) dogs. Nine of these dogs showed clinical improvement. The remaining dog was euthanized 10 months after partial cystectomy, as mentioned previously. In the six dogs with positive postoperative culture results, (four female and two male), all had less severe or less frequent clinical signs. The urachal anomaly was confirmed by results of histopathology in three of these six dogs.

## 4 DISCUSSION

The results of this retrospective study provide evidence that partial cystectomy in dogs with a urachal anomaly reduces the frequency or severity of clinical signs associated with LUTD and BUTI. Two‐thirds of dogs had no clinical recurrence of LUTD after a median follow‐up period of 22 months; 91% of dogs exhibited clinical improvement after partial cystectomy. Among the 25 dogs with confirmed preoperative BUTI, 22 showed clinical improvement after partial cystectomy. These results provide evidence that the removal of urachal anomalies plays a role in resolving LUTD, possibly by preventing or resolving either recurrent or persistent bacterial infections in the urinary bladder. All dogs with LUTD may benefit from the removal of these lesions because even those without confirmed positive urine culture may have had a BUTI at some point or at some other location (eg, in the bladder mucosa).^24^ Our results included a longer follow‐up and a larger cohort of dogs with LUTD and urachal anomalies than those reported for previous studies.

Repeated antibiotic treatments in dogs with recurrent BUTI have several disadvantages, including increased antimicrobial resistance. Therefore, antibiotic treatment is not effective to prevent urinary tract infection recurrence, so additional diagnostics are warranted to exclude predisposing causes, such as a urachal anomaly. In our cohort of dogs, cystoscopy seemed to be the most reliable technique to identify the lesion. However, other diagnostic methods, such as ultrasonographic examination of the abdomen, are also important to exclude other predisposing abnormalities that can be missed on cystoscopy alone, including pyelonephritis or prostatic disease.

Several types of urachal anomalies have been described in dogs. In our study, 97% of dogs had a macroscopic vesicourachal diverticulum, and only one dog had a urachal cyst, in line with previously published results.^3^ No microscopic diverticula were identified. Diverticula in the urinary bladder have been associated with urinary stasis and, hence, with recurrence of bacterial cystitis.^1^, ^4^ However, the clinical relevance of urachal cysts in relation to BUTI is unknown. In man, a urachal cyst is the most common presentation of urachal anomalies, but most of these are acquired^25^, ^26^; they usually present due to infection of the urachal cyst, although these cysts normally do not communicate with the bladder lumen.^27^ In man, an unresected urachal anomaly can progress to adenocarcinoma^28^, ^29^, ^30^; while this has been described once previously in a dog,^31^ it was not seen in this study.

The presence of urachal anomalies in our study was evenly distributed between sexes. This is in line with results of a previous study of dogs with subclinical urachal anomalies.^3^ We found a significantly younger median age at onset of clinical signs (12 months) in our study compared with a previous study (7.7 years).^2^ This difference may be because dogs with a congenital urachal anomaly in this study were predisposed for LUTD, resulting in an earlier onset of clinical signs compared with dogs with acquired LUTD.

Histopathology findings in dogs with urachal diverticula in this study were in line with findings in humans with benign bladder diverticula and consisted of changes in the mucosa and submucosa of the bladder wall but not the muscular layer.^3^, ^6^ Among the 28 dogs with excised bladder tissue examined by histopathology, the results for eight dogs revealed no evidence of a urachal remnant, even after evaluating additional histological tissue sections. Among these eight dogs, four had recurrence of clinical signs, and a urachal anomaly was diagnosed in three by cystoscopy only. In these latter three dogs, the anomaly may have been too small to identify during open surgical cystectomy, meaning that, during resection of the bladder apex, the actual anomaly was missed, leading to recurrence of clinical signs. After resection of the tissue, the diverticulum tends to collapse, and its localization may be difficult to identify after formaldehyde fixation, which could result in missing the urachal anomaly at histopathological examination. It would therefore be advisable to assess the size and ink the location of the anomaly before fixation. Unfortunately, five dogs had no histopathology of excised tissue performed. For these cases, a clear diagnosis of urachal anomaly was made preoperatively (one by ultrasonographic examination, two by positive‐contrast cystography, and two by cystoscopy). It is, however, strongly recommended that histopathology be performed on all excised bladder tissue, with or without a preoperative diagnosis based on advanced imaging.

In six male dogs and in one female dog, surgery was performed without a preoperative diagnosis of a urachal anomaly. Cystoscopy was not performed in five of six male dogs. For the remaining two dogs (one male and one female), cystoscopy was performed and failed to identify a diagnosis of urachal anomaly. In these seven dogs, surgical exploration of the cranial apex of the urinary bladder revealed an irregular serosal or mucosal surface and a difference in color or identification of a diverticula by close inspection of the mucosal surface during cystotomy. Partial cystectomy was performed, and results of histopathology confirmed the presence of a urachal anomaly in all seven cases. However, because the decision to perform a cystotomy in these dogs was subjective, some dogs with an anomaly may have been missed, and others may have undergone surgery. All dogs in which a cystotomy was performed in the present study showed either visible and/or histologic evidence of the presence of a urachal remnant.

Vesicourachal diverticula typically show herniation of the mucosa and submucosa through the muscular layer of the bladder wall.^3^, ^6^ Histopathology of microscopic vesicourachal diverticula is less well described but has been reported.^3^ No microscopic vesicourachal diverticula were identified in this cohort of dogs. These diverticula cannot be diagnosed with advanced diagnostic imaging or cystoscopy, and their clinical effect is unknown, but an association with lymphocytic infiltration in close proximity to the diverticulum or recurrent BUTI has been proposed.^3^, ^19^, ^32^ Although cystoscopy appeared to be most sensitive at detecting urachal diverticula, it may be challenging to detect these anomalies during surgical exploration and with histopathology. If the diverticulum is visible, cystoscopic removal of a small, intramural, diverticulum may be possible. Additional studies are required to determine whether blind partial cystectomy or cystoscopic removal of the urachal area in cases with no visible evidence of a urachal anomaly is justified.

One limitation of this retrospective study was the lack of a control group. A prospective clinical trial should include a control group of dogs with recurrent LUTD that are not treated surgically. At our institution, the standard treatment for these dogs is to have a partial cystectomy performed, so a randomized control group of sufficient dogs was not available. The standard advice to perform a partial cystectomy is based on limited scientific evidence.^4^, ^23^, ^30^, ^33^ Other limitations of this study were the variations in the preoperative and postoperative diagnostic procedures and urine cultures. Although our study appears to support the potential therapeutic effect of surgical resection of a urachal anomaly in dogs with LUTD, prospective randomized trials should be conducted to confirm the beneficial effect of this treatment.

Our results provide evidence that partial cystectomy is a beneficial additional treatment for dogs with a urachal anomaly and recurrent LUTD that may be associated with BUTI. Clinical signs were improved after surgery, and recurrence was reduced long term. However, confirmation by means of a prospective, standardized, and randomized trial is required.

## CONFLICT OF INTEREST

The authors declare no conflicts of interest related to this report.

## Supporting information


**Appendix S1**: Owner questionnaire

**Appendix S2**: Cultures and treatments

**Appendix S3**: Diagnostics
