# Supplementary material for: Resection of urachal anomalies in dogs with recurrent lower urinary tract disease
Source: Vet Surg. 2019 Aug 14;49(1):214–21. doi: 10.1111/vsu.13311 (PMC6973151; doi:10.1111/vsu.13311)
Supplement: Supplementary file 1 — Appendix S1: Owner questionnaire [file VSU-49-214-s001.pdf]

Owner questionnaire **Date:**

Name owner: **Name dog:**

Patient ID: **OR date:**

1. **Has your animal had any clinical signs of cystitis after surgery?**
   Examples:
   - **Frequent, small urination**
   - **Pain during urination**
   - **Abnormal urine (color, odor, etc)**

   Yes **No**

Additional notes:

……

……

2. **If yes, how long after surgery? (disregard the initial 2-week recovery period)**

   ………

3. **Did these clinical signs resolve spontaneously or did you take your dog to the vet?**

   Vet **Spontaneously**

4. **Did your vet do any diagnostics?**

   Yes:…………………………………. **No** **I don't know**

5. **Did your vet treat your dog for these signs of cystitis**

   Yes **No** **N/A**

6. **If yes, what medication /treatment and how long?**

……

……

7. **If yes, have clinical signs resolved after treatment?**

   Yes **No** **N/A**

8. **Has your animal had any clinical signs of cystitis after this?**

Yes **No**

9. **If yes, how often?**

1 **2** **3** **>3 times** **N/A**

Additional notes:

....

10. **What was the interval between episodes?**

....

....

10. How severe were the clinical signs related to cystitis before surgery? (0 is not severe, 5 is most severe)

0 1 2 3 4 5

11. If still present, how severe are clinical signs related to cystitis now? (0 is not severe, 5 is most severe)

0 1 2 3 4 5

12. Are you satisfied about the surgery and the immediate postoperative period? (0 is very unsatisfied, 5 is very satisfied)

0 1 2 3 4 5

13. Are you satisfied about the long-term effect of the surgery? (0 is very unsatisfied, 5 is very satisfied)

0 1 2 3 4 5

Additional notes:

....

....

....
