# Supplementary material for: Resection of urachal anomalies in dogs with recurrent lower urinary tract disease
Source: Vet Surg. 2019 Aug 14;49(1):214–21. doi: 10.1111/vsu.13311 (PMC6973151; doi:10.1111/vsu.13311)
Supplement: Supplementary file 2 — Appendix S2: Cultures and treatments [file VSU-49-214-s002.pdf]

Case #	Gender	Age at onset clinical signs	Duration of clinical signs at sx	Preoperative cultures	Preoperative antibiotics	Other preoperative treatment	Postoperative antibiotics	Other postoperative treatment	Postoperative cultures	Clinical improvement after surgery (0=no; 1= yes)	Preoperative clinical severity (0-5, 0=no clinical signs)	Postoperative clinical severity (0-5, 0=no clinical signs)	Long-term owner satisfaction (0=unsatisfied, 5=very satisfied)
1	M	4 years	2 months	None, clinical signs only	AC / enrofloxacine (duration unknown)		AC 10 days		N/A	1	4	0	5
2	M	2 1/2 months	4 weeks	None, clinical signs only	AC 7 days / TMPS 10 days		AC (duration unknown)		N/A	1	4	0	5
3	MN	4 months	21 months	None, clinical signs and urinalysis only. No positive culture, negative culture when on ab.	AC / enrofloxacine (duration unknown)		AC 10 days	Oxybutinin	E.coli 2 months after surgery	1	5	2	5
4	MN	4 years	3 months	E.coli x2	AC 5 days / AC 4 weeks / cefalexin 3 weeks		AC (duration unknown)		N/A	1	4	3	4
5	MN	3 1/2 years	3 years	None, clinical signs and urinalysis only.	AC / TMPS (duration unknown)	Oxybutinin	AC 7 days	Oxybutinin	N/A	1	5	0	5
6	MN	7 years	2 months	Negative culture x1, clinical signs and urinalysis only.		Carbachol		Oxybutinin	N/A	1	4	0	4
7	FS	2 1/2 years	2 years	Proteus mirabilis x3	AC 10 days, AC 2 weeks, AC 4 weeks, AC 6 weeks		AC 14 days		Negative culture 1 month after surgery	1	3	0	5
8	FS	2 months	2 years	Proteus mirabilis x2	AC 5 days, AC 7 days, AC 10 days, AC 3 weeks / enrofloxacine 6 weeks		Enrofloxacine 4 weeks		N/A	1	4	0	5
9	F	9 months	1 1/2 years	E.coli x1; Proteus mirabilis x1	AC 3 weeks / unknown antibiotic (type and duration)		AC 6 days	Oxybutinin	Urinalysis negative 1 month after surgery	1	5	0	5
10	MN	10 years	2 years	E.coli x1, Proteus mirabilis x1, Strep spp x2, unknown culture x1	AC 5 days , AC 7 days, AC 7 days AC 5 days / TMPS (unknown duration) / enrofloxacine (unknown duration)		AC 7 days		N/A	0	5	5	0
11	M	3 1/2 months	3 months	Negative culture x3, clinical signs and urinalysis only.	AC 7 days	Oxybutinin	AC 10 days	Oxybutinin	Negative culture 3 and 6 months after surgery	1	5	0	5
12	F	3 months	1 1/2 years	Positive culture x2, type unknown	AC 2 courses (unknown duration)			Oxybutinin	N/A	0	4	4	0
13	MN	7 years	3 months	E.coli x3, negative culture x2 (on antibiotics)	AC 7 days, AC 10 days, AC 2 weeks, AC 3 weeks, AC 3 weeks	Phenylpropanolamin	AC 7 days		N/A	1	3	0	5
14	FS	4 years	3 years	Strep spp x1	AC 10 days, AC 2 weeks / doxycycline 10 days		AC 10 days	Oxybutinin	N/A	1	4	1	5
15	MN	5 months	7 months	E.coli x2, negative culture x1 (on antibiotics)	AC long course (duration unknown) / TMPS long course (duration unknown) / enrofloxacine (duration unknown)	Oxybutinin	AC 2 weeks		Negative culture 1 and 11 months afther surgery	1	5	0	5
16	FS	8 years	7 years	Staphylococcus pseudointermedius x1, negative culture x1 (on antibiotics)	AC 10 days, AC 3 weeks, AC 3 weeks / TMPS 3 weeks, TMPS 2 weeks (+ 4 weeks postoperatively)		TMPS 4 weeks		None	1	4	0	5
17	MN	3 months	3 years	E.coli x2, negative culture x1 (on antibiotics)	AC 2 weeks		AC 7 days		Negative culture 1 month after surgery	0	5	5	0
18	FS	1 year	8 years	E.coli x1, Proteus mirabilis x1, unknown culture x1, negative culture x1 (on antibiotics)	AC 10 days, AC (unknown duration) / TMPS 6 weeks		AC 7 days		Streptococcus spp 1 month after surgery, negative culture 2 months after surgery	1	5	3	3
19	M	6 years	6 months	Staphylococcus pseudointermedius x3, MRSI x1, negative culture x2 (1x on antibiotics)	AC 2 weeks, AC 2 weeks, AC 3 weeks, AC 2 weeks, AC 2 weeks (started preoperatively)		AC 10 days (started preoperatively)		Negative culture 1 and 2 months after surgery, MRSP 6 months after surgery, negative culture 7 and 8 months after surgery	1	5	2	4
20	FS	5 1/2 years	7 years	Proteus mirabilis x2, negative culture x2 (1x on antibiotics)	AC 7 days , AC 2 weeks		AC 10 days		N/A	1	5	3	5
21	F	2 months	2 months	E.coli x2, negative culture x1 (on antibiotics)	AC 8 days, AC 6 weeks		AC 2 weeks (started preoperatively)		Negative culture 1 month after surgery	1	4	0	5
22	F	9 months	14 months	Proteus mirabilis x1, negative culture x1 (on antibiotics)	AC 7 days, AC short course (duration unknown), AC long course (duration unknown) / enrofloxacine 2 weeks, enrofloxacine 7 days		Enrofloxacine 7 days (started preoperatively)		Proteus mirabilis 10 months after surgery, negative culture 11, 12 and 13 months after surgery	1	5	4	N/A
23	MN	8 years	1 year	E.coli x2, negative culture x1 (on antibiotics)	AC 7 days, AC 7 days, AC 7 days, AC 4 weeks, AC 4 weeks		AC 7 days (started preoperatively)		Negative culture 1 month after surgery	1	4	0	5
24	M	5 weeks	2 months	None, clinical signs and urinalysis only (2x bacteria seen in sediment on urinalysis)	AC 5 days, AC 10 days, AC 7 days		AC 7 days		N/A	1	4	0	5
25	F	2 months	4 months	E.coli 2x	AC 10 days, AC 3 weeks / enrofloxacine 4 weeks		Enrofloxacine 3 weeks (started preoperatively)		Negative 2 weeks after surgery (on enrofloxacine)	1	5	0	5
26	FS	5 years	1 year	E.coli 1x, negative culture x2	AC 10 days, AC 2 weeks		AC 2 weeks		Urinalysis negative 1 month after surgery	1	3	0	5
27	F	3 months	4 months	E.coli x3, negative culture x1 (on antibiotics)	AC 7 days, AC 10 days, AC 10 days, AC 4 weeks		AC 10 days		E.coli 1 month after surgery (subclinical)	1	5	3	3
28	M	8 years	5 months	Staphylococcus intermedius x2, Staphylococcus pseudointermedius x2	AC 3 weeks, AC 4 weeks, AC 4 weeks / TMPS 3 weeks / doxycycline 3 weeks		AC 10 days		N/A	1	3	0	5
29	MN	"always"	"always"	None, clinical signs and urinalysis only	AC (unknown duration)		AC 2 weeks (started preoperatively)		Negative culture 2 months after surgery	1	4	0	5
30	FS	4 months	8 months	E.coli x1, positive culture x3, type unknown, negative culture x1 (on antibiotics)	AC 7 days, AC 6 weeks, AC 5 days / TMPS2 weeks, TMPS 4 weeks / 7dg cefalexine 7 days, cefalexine 10 days / metronidazole 5 days		AC 10 days		Negative culture 1 month after surgery	1	5	0	5
31	F	2 months	4 months	E.coli x2, negative culture x1 (on antibiotics)	AC 10 days, AC 10 days, AC 2 weeks		AC 2 weeks		N/A	1	5	0	5

32	M	7 weeks	7 weeks	E.coli x2	AC 5 days, AC 2 weeks, AC started preoperatively	Desmopressine	AC 2 weeks (started preoperatively)	Desmopressine	Negative culture 1 month after surgery	1	3	0	5
33	F	5 months	7months	E.coli x1	AC 4 weeks (started preoperatively) / TMPS 7 days, TMPS 7 days, TMPS 10 days		AC 2 weeks (started preoperatively)		Proteus mirabilis 6 months after surgery	1	5	4	4
AC = amoxicilline clavulanic acid													
TMPS = trimethoprim sulfamethoxazole													
