# Supplementary material for: Resection of urachal anomalies in dogs with recurrent lower urinary tract disease
Source: Vet Surg. 2019 Aug 14;49(1):214–21. doi: 10.1111/vsu.13311 (PMC6973151; doi:10.1111/vsu.13311)
Supplement: Supplementary file 3 — Appendix S3: Diagnostics [file VSU-49-214-s003.pdf]

Case #	Breed	Gender	Ultrasound	Cystography	Cystoscopy	Exploratory cystotomy	Histopathology
1	English Cocker Spaniel	M	**Positive**	Not performed	Not performed	Not performed	**Positive**
2	Staffordshire Bullterrier	M	**Positive**	Not performed	Not performed	Not performed	**Positive**
24	Cane Corso	M	**Positive**	Not performed	Not performed	Not performed	Not performed
32	Weimaraner	M	**Positive**	Not performed	Not performed	Not performed	False negative
31	Rhodesian Ridgeback	F	**Positive**	Not performed	**Positive**	Not performed	**Positive**
3	Newfoundland	MN	**Positive**	**Positive**	Not performed	Not performed	Not performed
17	Labrador Retriever	MN	False negative	False negative	False negative	Positive	**Positive**
13	German Shepherd	MN	False negative	False negative	Not performed	Positive	**Positive**
12	English Springer Spanniel	F	False negative	False negative	**Positive**	Not performed	Not performed
15	Rottweiler	MN	False negative	False negative	**Positive**	Not performed	**Positive**
21	Irish Terrier	F	False negative	False negative	**Positive**	Not performed	Not performed
16	Dutch Sheepdog	FS	False negative	Not performed	False negative	Positive	**Positive**
4	Great Dane	MN	False negative	Not performed	Not performed	Positive	**Positive**
19	English Cocker Spaniel	M	False negative	Not performed	Not performed	Positive	**Positive**
28	English Cocker Spaniel	M	False negative	Not performed	Not performed	Positive	**Positive**
29	Boxer	MN	False negative	Not performed	Not performed	Positive	**Positive**
7	Cavalier King Charles Spaniel	FS	False negative	Not performed	**Positive**	Not performed	**Positive**
9	ChowChow	F	False negative	Not performed	**Positive**	Not performed	**Positive**
14	Labradoodle	FS	False negative	Not performed	**Positive**	Not performed	**Positive**
18	French Bulldog	FS	False negative	Not performed	**Positive**	Not performed	False negative
20	Petit Bassin Griffon	FS	False negative	Not performed	**Positive**	Not performed	False negative
22	Shepherd cross	F	False negative	Not performed	**Positive**	Not performed	**Positive**
23	Airedale Terrier	MN	False negative	Not performed	**Positive**	Not performed	**Positive**
25	Labradoodle	F	False negative	Not performed	**Positive**	Not performed	False negative
26	Cavalier King Charles Spaniel	FS	False negative	Not performed	**Positive**	Not performed	False negative
27	Swiss Mountain Dog	F	False negative	Not performed	**Positive**	Not performed	**Positive**
30	Boxer	FS	False negative	Not performed	**Positive**	Not performed	**Positive**
33	Bearded Collie	F	False negative	Not performed	**Positive**	Not performed	False negative
5	Bernese Mountain Dog	MN	False negative	**Positive**	Not performed	Not performed	**Positive**
6	Chihuahua	MN	False negative	**Positive**	Not performed	Not performed	False negative
8	Bernese Mountain Dog	FS	False negative	**Positive**	Not performed	Not performed	Not performed
10	Staffordshire Bullterrier	MN	False negative	**Positive**	Not performed	Not performed	False negative
11	Bernese Mountain Dog	M	False negative	**Positive**	Not performed	Not performed	**Positive**
